# Decision-making process in the pre-dialysis CKD patients: do anxiety, stress and depression matter?

**DOI:** 10.1186/s12882-018-0896-3

**Published:** 2018-04-27

**Authors:** Cicero Italo L. Bezerra, Bruno C. Silva, Rosilene M. Elias

**Affiliations:** 1 Nephrology, Hospital das Clinicas HCFMUSP, Universidade de Sao Paulo, São Paulo, Brazil; 2 Universidade Nove de Julho (UNINOVE), São Paulo, Brazil

**Keywords:** Anxiety, Stress, Depression, Peritoneal dialysis, Hemodialysis

## Abstract

**Background:**

The transition from pre-dialysis chronic kidney disease (CKD) to renal replacement therapy (RRT) is a stressful event. Anxiety, depression and stress are frequent conditions in this population, and might play a role on the choice of dialysis modality.

**Methods:**

This is a prospective study that included stages 4-5 CKD patients during a dialysis multi-disciplinary education program. Demographic, clinical, and laboratory data were evaluated. Hospital Anxiety and Depression Scale and a Perceived Stress Scale assessed levels of anxiety, depression and stress, respectively.

**Results:**

A total of 67 from 190 recruited patients were included (59 ± 15 years, 54% males). Comparing patients who chose peritoneal dialysis (PD) and hemodialysis (HD), there were no differences on anxiety (*p* = 0.55), and depression scores (*p* = 0.467), and stress (*p* = 0.854). Anxious (*p* = 0.007) and depressive (*p* = 0.030) patients presented lower levels of phosphate than those not affected. There was a significant correlation (*p* < 0.0001) between anxiety and depression scores (*R*^2^ = 0.573), anxiety and stress scores (*R*^2^ = 0.542), depression and stress scores (*R*^2^ = 0.514). At the end of study, 29.8% of patients had already started on dialysis, and scores of anxiety, depression and stress reduced significantly (all *p* values < 0.0001), from 5.9 ± 3.3 to 1.8 ± 1.8, from 7.7 ± 4.0 to 3.8 ± 2.9 and from 28.6 ± 7.8 to 10.0 ± 6.2, respectively, regardless of which therapy was chosen.

**Conclusion:**

Depression, anxiety and perceived stress during final stages of CKD do not seem to be related to the choice of dialysis therapy and tend to decrease after dialysis initiation.

## Background

Depression and anxiety are frequent comorbid disorders among chronic kidney disease (CKD) patients, with estimated prevalence of approximately 25% in this population, [1] and are associated with worse outcomes, such as progression to end-stage renal disease (ESRD) and mortality [2–5]. The transition from predialysis management to renal replacement therapy (RRT) is a stressful event in the course of CKD, leading to challenges and decisions that might increase their susceptibility to anxiety, mood disorders or even exacerbate psychological issues that already exist [6].

Choosing an RRT might be a dilemma for both patients and nephrologists since different options of RRT are available for CKD patients. Preemptive kidney transplantation, which might lead to higher life expectancy and quality of life [7], is an option for these patients if a living donor is available. Therefore, peritoneal dialysis (PD) and hemodialysis (HD) are the most common modalities offered to patients requiring RRT.

The RRT decision-making process is challenging; nephrologists should provide education and support to help choose the dialysis modality that best reflects the patient’s personal values and lifestyle [8, 9]. Nevertheless, frequently only clinical, social and economic variables are taken into account for such decision, while mental health is often neglected.

Despite the clear association between mood disorders and CKD, there is limited data regarding the influence of such disorders on the decision-making process in pre-dialysis CKD patients. Given the complexity of the process of choosing a dialysis modality, the goal of this study is to determine the extent of which the severity of anxiety, depression, and stress among pre-dialytic patients can interfere with the choice of the RRT method.

## Methods

This was a prospective study that recruited patients from a tertiary academic hospital in Sao Paulo, Brazil, during a dialysis education program (DEP), given by a multi-disciplinary team of nephrologist, nurse, psychologist, dietician and social worker. The eligibility criteria were adult pre-dialysis CKD (stages 4 and 5) patients who attended the DEP and agreed to participate in the study by signing the Informed Consent Form. Demographic, clinical, and laboratory data were obtained from medical records. At study entry, patients answered the Portuguese version of the self-administered Hospital Anxiety and Depression Scale (HADS) and Perceived Stress Scale (PSS) to assess their scores of anxiety, depression, and stress, respectively, as described below. Participants also filled in a survey with questions about the reasons why they decided for a particular dialysis modality.

### Hospital anxiety and depression scale (HADS)

This tool has 14 items that evaluate anxiety and depression severity, yet excluding somatic symptoms to prevent bias from physical disorders. Scores ≥9 establish the diagnosis of anxiety and depression [10].

### Perceived stress scale (PSS)

This scale is composed of 14 items that assess how individuals perceive stressful situations. The total sum of the scores can reach 56 points. Higher scores reflect greater amounts of perceived stress [11].

### Statistical analysis

Continuous data were presented as mean and SD or median (25, 75) according to data distribution, as appropriate. Categorical data were displayed as N and percentage. Comparison between the groups of patients who chose PD or HD was performed using the independent t-test or the alternative Mann–Whitney U-test, as appropriate. A *p*-value < 0.05 was considered statistically significant.

## Results

A total of 190 adult patients who had been diagnosed with Stage 4 or 5 CKD and were assigned to DEP were randomly selected and asked to participate in the research. Out of the 190 candidates, a total of 69 patients agreed to participate in the study. Two participants were excluded from the study due to a lack of final decision on RRT modality. Therefore, a total sample of 67 patients was included. From this population, 22 chose PD and 45 preferred HD as future RRT option.

Patient’s demographic and social characteristics are shown in Table 1. PD and HD groups did not differ regarding any demographic, clinical and biochemical characteristic. Mean age of the patients was 59 ± 15 years, similar between PD and HD groups. The sample is comprised of 54% males, 59% white, 56% married patients. Almost 78% of the sample had an education level ending before or at ninth grade, particularly in the group of patients who chose Peritoneal Dialysis – PD (82%) when compared to the group who elected Hemodialysis – HD (71%). The leading causes of CKD were Nephrosclerosis and Diabetic Nephropathy.

**Table 1:** Baseline patient characteristics

	Total	Dialysis modality		
		PD	HD	*p*
Characteristic	(*N* = 67)	(*N* = 22)	(*N* = 45)	
Age, years	59 ± 15	59 ± 17	58 ± 14	NS
Male, %	53.7	54.5	53.3	NS
White, %	63.8	71.4	59.5	NS
Primary renal disease, %				
Nephrosclerosis	30.8	22.7	34.9	NS
Diabetic Nephropathy	24.6	22.7	25.6	
Glomerulonephritis	12.3	9.1	14.0	
Others and unknown	32.3	45.5	25.5	
Comorbidities, %				
Diabetes	41.5	40.9	41.9	NS
Hypertension	81.5	77.3	83.7	NS
Myocardial infarction	10.6	4.5	13.6	NS
Stroke	4.5	0	6.8	NS
Congestive heart failure	15.2	9.1	18.2	NS
Atrial Fibrillation	7.6	9.1	6.8	NS
Peripheral arterial disease	12.1	9.1	13.6	NS
COPD	6.1	0	9.1	NS
Married, %	60.9	63.6	59.5	NS
Higher education level, %				
Before ninth grade	77.8	81.8	75.6	NS
Up to high school	15.9	18.2	14.6	
College/University	6.3	0	9.8	
Laboratory values				
eGFR, (ml/kg/1.73m^2^)	16 ± 7	14 ± 6	17 ± 7	NS
Creatinine (mg/dL)	4.1 ± 1.7	4.4 ± 1.6	4.0 ± 1.8	NS
Urea (mg/dL)	124 ± 50	127 ± 47	123 ± 52	NS
Potassium (mmol/L)	4.7 ± 0.7	4.7 ± 0.7	4.7 ± 0.7	NS
Albumin (g/dL)	4.0 ± 0.6	4.1 ± 0.6	4.0 ± 0.6	NS
Phosphorus (mg/dL)	5.0 ± 2.1	5.3 ± 2.4	4.9 ± 2.0	NS
PTH (pg/mL)	177 (107, 292)	215 (112, 265)	153 (98, 298)	NS
Total Calcium (mg/dL)	9.2 ± 0.8	9.0 ± 1.0	9.3 ± 0.7	NS
25-hydroxyvitamin D (ng/mL)	23.5 ± 11.3	20.8 ± 14	25.2 ± 9.2	NS
Bicarbonate (mmol/L)	21.4 ± 6.3	20.3 ± 8.5	22.1± 4.6	NS
Alkaline phosphatase (UI/L)	103 ± 61	114 ± 44	98 ± 69	NS
Iron (μg/ml)	68.6 ± 28.8	75 ± 41	65 ± 20	NS
Ferritin (ng/ml)	176 (79, 394)	121 (65, 368)	209 (92, 408)	NS
Hemoglobin (g/L)	11.0 ± 2.8	10.9 ± 3.1	11.1 ± 2.6	NS

*eGFR* estimated glomerular filtration rate, *COPD* chronic obstructive pulmonary disease, *NS* not significant

Patients were asked about particular reasons that made them chose PD or HD. Family and work issues accounted 32.8% and 7.5%, respectively. Patients who chose PD over HD were more likely to select work issue (9.1% vs. 6.7%).

**Fig. 1:**
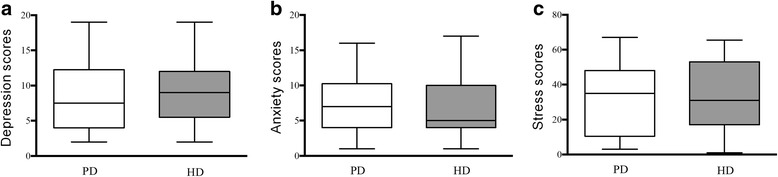
Depression (**a**), Anxiety (**b**), and Perceived stress (**c**) levels comparison between peritoneal dialysis (PD) and hemodialysis (HD) groups. White bars represent PD group and grey bars represent HD group. Box and whiskers represent median and range

Table 2 shows median scores of anxiety, depression and stress scores in the entire group as well as according to the dialysis modality chosen. The median of Anxiety scores was 6, ranging from 1 to 17, which was not different when comparing PD and HD groups (Fig. 1a). Patients classified as anxious corresponded to 45% of the PD group and 29% of the HD group (*p* = 0.180). There was no difference between anxious and non-anxious patients regarding gender (*p* = 0.483), race (*p* = 0.277), education level (*p* = 0.305), and marital status (*p* = 0.617). Anxious patients presented lower levels of phosphate than non-anxious subjects (4.3 ± 0.8 vs. 5.4 ± 2.5, *p* = 0.007).

**Table 2:** Levels of Anxiety, depression and stress according to chosen dialysis modality

	Total	Dialysis modality		
		PD	HD	*p*
Scores	(*N* = 67)	(*N* = 22)	(*N* = 45)	
Anxiety	6 (4, 10)	7 (4, 10)	5 (4, 10)	NS
Depression	9 (5, 12)	7.5 (4, 12)	9 (6, 12)	NS
Stress	30 (21, 35)	30 (20, 34)	29 (22, 36)	NS

Values are expressed as median (25, 75)

*NS* not significant

Median depression scores were 9, ranging from 2 to 19. Median depression scores in PD and HD groups were not significantly different (Table 2 and Fig. 1b). Using a cut-off value of ≥9, we found a similar prevalence of depression in HD and PD groups of 58% and 41%, respectively (*p* = 0.194). Depressive patients presented lower levels of phosphate (4.5 ± 1.2 vs. 5.6 ± 2.7, *p* = 0.030), and a tendency to higher PTH levels [180 (127, 305) vs. 153 (98, 248) pg/ml, *p* = 0.102].

Levels of stress did not differ significantly between groups of patients who elected PD and HD, as shown in Table 2 while median values for the entire population were 30 (21, 35). As seen in Fig. 1c, scores of stress varied from 18 to 47 in PD group, and from 17 to 42 in HD group.

**Fig. 2:**
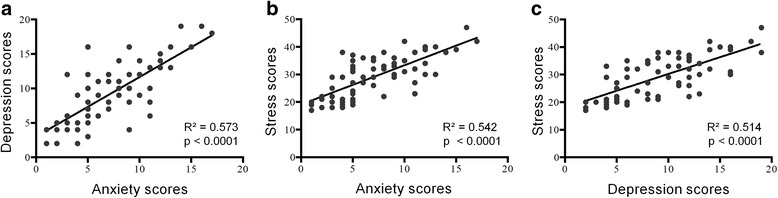
Correlations between anxiety and depression scores (**a**), anxiety and stress scores (**b**), and depression and stress scores (**c**)

When analyzing the entire population, anxiety and depression scores show a positive and strong correlation (*R*^2^ = 0.573; *p* < 0.0001; Fig. 2a). There was a correlation between scores of anxiety and stress (*R*^2^ = 0.542; *p* < 0.0001; Fig. 2b) and also between depression and stress scores (R^2^ = 0.514; *p* < 0.0001; Fig. 2c).

At the end of the study, 20 patients (30%) had already started on dialysis. From this sample, eight patients chose PD as preferred therapy, but only five actually started PD and the three remaining started HD. All patients who had chosen HD actually started it. We have applied all scales within the first 3 months after dialysis initiation. A subanalysis of this sample revealed that scores of anxiety, depression, and stress reduced significantly regardless of which therapy was chosen (Table 3).

**Table 3:** Levels of Anxiety, depression and stress before in after dialysis initiation

	Before dialysis initiation (*N* = 20)	After dialysis initiation (*N* = 20)	*p*
Anxiety	5.9 ± 3.4	1.8 ± 1.8	0.0001
Depression	7.6 ± 4.0	3.8 ± 2.9	0.0001
Stress	28.6 ± 7.8	10.0 ± 6.2	0.0001

Values are mean ± SD

## Discussion

This study aimed to understand if symptoms of depression, anxiety or stress in patients with CKD have any impact on the choice of dialysis therapy. We found that the prevalence of both anxiety and depression is high in this subset of patients, regardless of chosen therapy, and that perceived stress is associated with both conditions. Nevertheless, after starting dialysis, there was a significant reduction in overall depression, anxiety, and stress scores.

In CKD patients, depression has been implicated not only with poor outcomes and low adherence to general medical treatment [12] but also with anxiety and low quality of life [13]. However, the major concern of healthcare providers is the association of depression with higher risk of initiation of dialysis, hospitalization, and death in this population [2–5]. This is of huge clinical relevance since the prevalence of depression across different populations ranges from 7 to 47% [13–16]. In the current study, we found a point prevalence of depression of 58% and 41% in CKD patients who chose HD and PD as dialysis methods, respectively. Such finding represents a novelty since the impact of depression on the choice of dialysis method is still unclear. Additionally, depression was associated with both anxiety and perceived stress.

Anxiety symptoms often co-occur with depression not only in the general population but also in patients undergoing dialysis [1]. Few studies have addressed anxiety in CKD patients and its prevalence across cohorts varies from 27 to 31% [3, 13], which is similar to what we found. So far, anxiety seems to play a role in strengthening the negative association between depression and quality of life in dialysis patients, thus reducing the ability to cope with this condition [1].

We found higher levels of perceived stress in comparison to a US cohort, which described a mean PSS score of 5.6 in 65 patients with CKD 4 and 5 [17]. Recently, higher PSS scores were associated with increased mortality in patients with multiple comorbidities [18]. Since stress could be a modifiable risk factor, possibly associated with lifestyle choices, diagnosis and management of this condition could improve management of CKD care.

Regardless of whether or not the patient has depressive or anxious symptoms during the course of CKD, it does not seem to influence the choice of dialysis. Supposedly, it would be convenient for a depressive/anxious patient not to choose PD, since such method requires self-care. The opposed finding in this study carries out an important message to health care providers since the presence of these symptoms does not necessarily indicate an alternative with less need for self-care.

We also found a correlation between depression and anxiety with lower phosphate levels. This is not entirely new since it has been previously demonstrated [19, 20], although in non-CKD population. In the general population, this correlation can be explained by alkalosis caused by hyperventilation, which is commonly found in patients with anxiety and depression [21]. However, regarding pre-dialysis CKD patients, low levels of phosphate might reflect malnutrition. Serum calcium and hormones involved in calcium homeostasis are less likely to be involved in this process [19]. Accordingly, we did not find any associations between depression or anxiety and PTH, vitamin D or calcium levels.

Another interesting finding was the predilection for HD rather than PD. Patients had no limitations in choosing between both therapies. We believe that this particular preference for HD in Brazil is due to cultural issues, as self-care is the main barrier to choosing PD. Many patients prefer a professional person to take care of dialysis therapy, even though it might lead to less freedom to work or travel.

This study has some limitations, as comprised a small sample size and individuals were included from a single center. Additionally, results can be biased since patients who agreed to participate could be more motivated than those who refused to participate. Additionally, cut-off values for diagnosis of anxiety/depression in the CKD population are still under debate. The current study has also some strength: to the best of our knowledge, this is the first study that assessed the impact of depression, anxiety, and stress in an RRT decision-making process. Furthermore, we have demonstrated that these symptoms improve after dialysis initiation. The relationship between hypophosphatemia and depressive/anxious symptoms in the setting of CKD deserves further investigation.

## Conclusions

In summary, depression, anxiety and perceived stress during final stages of CKD do not seem to influence the choice of dialysis therapy. Additionally, after starting dialysis, it is observed a significant reduction in overall depression, anxiety, and stress scores.

## Abbreviations

CKD: Chronic kidney disease
DEP: Dialysis education program
HADS: Hospital anxiety and depression scale
HD: Hemodialysis
PD: Peritoneal dialysis
PSS: Perceived stress scale
RRT: Renal replacement therapy
